# Nationwide Trends in Cardiovascular and Cerebrovascular Diseases and Outcomes Among Young Adults (18-39 years) Hospitalized With Comorbid Depression

**DOI:** 10.7759/cureus.10640

**Published:** 2020-09-24

**Authors:** Rupak Desai, Vijaya Lakhsmi Valaparla, Hee Kong Fong, Zainab J Gandhi, Yash Varma, Kaksha Varma, Mir Z Ali Khan, Bashar Dawood, Virmitra Desai, Sejal Savani, Rajkumar Doshi, Zeeshan Mansuri

**Affiliations:** 1 Cardiology, Atlanta Veterans Affairs Medical Center, Decatur, USA; 2 Neurology, Glenn Biggs Institute for Alzheimer's and Neurodegenerative Diseases, University of Texas Health Science Center at San Antonio, San Antonio, USA; 3 Cardiovascular Medicine, University of California Davis Medical Center, Sacramento, USA; 4 Medicine, C.U. Shah Medical College, Surendranagar, IND; 5 Internal Medicine, Government Medical College, Bhavnagar, IND; 6 Psychiatry, Center for Psychiatric Neuroscience, The Zucker Hillside Hospital, Glen Oaks, USA; 7 Internal Medicine, Mercy Catholic Medical Center, Darby, USA; 8 Internal Medicine, St. Vincent's Hospital, Jacksonville, USA; 9 Public Health, University of North Texas Health Science Center Fortworth, Fortworth, USA; 10 Public Health, New York University, New York, USA; 11 Internal Medicine, University of Nevada Reno School of Medicine, Reno, USA; 12 Psychiatry, Texas Tech University Health Sciences Center at Odessa, Midland, USA

**Keywords:** young adults, depression, mental health, cardiovascular disease, myocardial infarction, arrhythmia, stroke, mortality, trends

## Abstract

Background

Modern-day studies that assess temporal trends in cardiovascular and cerebrovascular events (CCE) and outcomes among the young population in the United States (US) with depression remain limited.

Methods

We compared baseline demographics, comorbidities, all-cause mortality, acute myocardial infarction (AMI), percutaneous coronary interventions (PCI), arrhythmia, stroke, and venous thromboembolism (VTE) among hospitalized young adults (18-39 years) with vs. without depression using the National Inpatient Sample (NIS) from 2007 to 2014.

Results

A total of 3,575,275 patients out of 63,020,008 hospitalized young adults had comorbid depression (5.7%; median 31 years, 71.3% females). The depressed cohort more often comprised of older, white, male, and non-electively admitted patients. Higher rates of comorbidities, all-cause mortality, PCI, arrhythmia, VTE, and stroke were observed among the depressed cohort. The rising trend in all-cause mortality was observed among the depressed against a stable trend in the non-depressed. The prevalence of AMI remained stable among depressed with consistent upsurges in arrhythmia and stroke. Those with depression had extended hospital stay, higher hospitalization charges, and were more often transferred to other facilities or discharged against advice.

Conclusions

Rising trends of inpatient mortality, CCE, and higher resource utilization among young adults with depression are concerning and warrants a multidisciplinary approach to improve quality of life and outcomes.

## Introduction

Depression and cardiovascular disease (CVD) are known to be the leading causes of disability worldwide [1]. The association between depression and CVD has been established in previous studies and is found across the ages. Depression has been found to be an independent risk factor of mortality and re-hospitalization or reinfarction in patients with known CVD in prior etiological and longitudinal studies; however, contemporary large-scale data remains limited [2-5]. Biologically plausible mechanisms such as autonomic nervous system dysfunction, altered platelet receptor functioning, and pro-inflammatory cytokines, through which depression can influence the etiology and outcome in CVD, have been studied [6-8]. Depression can lead to non-compliance to medications and health-promoting behaviors like exercise and a healthy diet. The relationship of a sedentary lifestyle and lack of exercise among patients with mental health disorders and possible health consequences are well discussed in the literature [9-11]. Diagnostic challenges and under-treatment are highly concerning when depression presents as a comorbidity with cardiovascular conditions.

Clinical depression in young adults is associated with increased risk of CVD risk factors like uncontrolled blood pressure, smoking, alcohol consumption, and obesity, as well as a lower quality of life and an increased risk of coronary heart disease [12-17]. However, modern-day large-scale studies in the United States (US) that assess the relationship of depression in young adults with CVD and outcomes including mortality with an emphasis on shifting trends remain scarce. The current study, with its large nationally representative sample size, aims at comparing trends in the prevalence of CVD and in-hospital outcomes in young adults with and without depression from 2007 to 2014.

## Materials and methods

This study was carried out based on the inpatient data of the National Inpatient Sample (NIS) from the Agency for Healthcare Research and Quality-supported Healthcare Cost Utilization Project from 2007 to 2014 [18]. NIS consists of a 20% stratified sample of all nonfederal community hospitals in the US exclusive of acute and rehabilitation care facilities, and when weighted, it represents 95% of the US population. The NIS data outlines patients’ demographics, up to 25 diagnoses and 15 procedures, hospital characteristics, and comorbidities with relevant International Classification of Diseases Clinical Modification, 9th Revision (ICD-9 CM) codes [5,19]. The current study was exempt from the institutional review board approval owing to the publicly available and de-identified nature of the datasets.

The study cohort consisted of hospitalized young adults (18-39 years) with comorbid depression (ICD-9 CM codes: 296.2x, 296.3x, 300.4, and 311). The primary outcomes were temporal trends in the frequencies of all-cause mortality, acute myocardial infarction (AMI), percutaneous coronary intervention (PCI), arrhythmia, stroke, and venous thromboembolism (VTE). Secondary outcomes were an evaluation of baseline demographics, comorbid conditions, disposition, the mean length of stay (LOS, days), and hospital charges. Coexistent cardiopulmonary conditions and procedures were identified with ICD-9 CM codes as detailed earlier [19-21].

SPSS Statistics v22 (IBM Corp., Armonk, New York) was used for all analyses. Continuous variables were described as mean or median as appropriate, and categorical variables as frequencies. Student’s t-test or Mann-Whitney nonparametric test were used to compare continuous variables, and Pearson chi-square was utilized to compare categorical variables. A linear-by-linear association test was used for trend analyses. All statistical tests were two-sided and a p-value of <0.05 was considered statistically significant.

## Results

From 2007 to 2014, there were about 63 million total hospitalizations among young adults (18-39 years), of which 3.6 million (5.7%) patients had clinical depression (Table 1A). The group with comorbid depression had a higher percentage of male patients compared to that without depression (28.7% vs. 22.4%; p<0.001). However, both groups predominantly consisted of females (71.3% depression vs. 77.6% no-depression). Of note, 71.5% of patients with depression were white, which was higher compared to the non-depression group (54.1%; p<0.001). African-Americans and Hispanics constituted 17.9% and 18.9% respectively of the non-depression group, which was slightly higher compared to the depression group. Both groups consisted of equivalent enrollees with private and Medicaid insurance and had comparable median household income. The cohort with depression had higher non-elective admissions (75.2% vs. 65.4%; p<0.001), and about half of the patients in both the groups had admissions in urban-teaching hospitals (51.8% and 53.8% respectively). Midwestern hospitals had the highest number of admissions in both groups (38.9% and 35.4% respectively). The depressed group had significantly higher comorbidities including alcohol abuse (7.0% vs. 3.6%), smoking (30.5% vs. 13.1%), congestive heart failure (CHF) (1.3% vs. 0.6%), diabetes (7.0% vs. 3.3%), diabetes with chronic complications (2.2% vs. 0.8%), dyslipidemia (7.1% vs. 2.5%), drug abuse (13.5% vs. 6.7%), hypertension (18.9% vs. 8.1%), obesity (12.4% vs 6.9%), peripheral vascular disorders (0.6% vs. 0.3%), pulmonary circulation disorders (0.8% vs. 0.4%), renal failure (3.2% vs. 1.5%), valvular heart disease (1.2% vs. 0.6%), and previous myocardial infarction/revascularization (1.0% vs. 0.4%) (all p<0.001) (Table 1B).

**Table 1:** Baseline characteristics and comorbidities in hospitalizations among young adults (18-39 years) with vs. without depression ^1^P-value of <0.05 indicates statistical significance IQR: interquartile range; HMO: health maintenance organization; MI: myocardial infarction; PCI: percutaneous coronary intervention; CABG: coronary artery bypass grafting

Variables	No depression (n=59,444,733)	Depression (n=3,575,275)	Overall (n=63,020,008)	P-value^1^
A. Demographics				
Age in years at admission, median (IQR)	29 (24-34)	31 (26-36)	29 (24-33)	<0.001
Sex				<0.001
Male	22.4%	28.7%	22.8%	
Female	77.6%	71.3%	77.2%	
Race				<0.001
White	54.1%	71.5%	55.1%	
African American	17.9%	14.1%	17.7%	
Hispanic	18.9%	9.6%	18.3%	
Asian or Pacific Islander	3.7%	1.1%	3.6%	
Native American	0.9%	0.9%	0.9%	
Others	4.5%	2.8%	4.4%	
Median household income, national quartile for patients' ZIP Code				<0.001
0-25^th^	30.1%	29.5%	30.1%	
26-50^th^	25.7%	27.4%	25.8%	
51-75^th^	23.8%	24.3%	23.9%	
76-100^th^	20.4%	18.8%	20.3%	
Hospital-level characteristics				
Primary expected payer				<0.001
Medicare	4.6%	11.2%	5.0%	
Medicaid	35.8%	33.5%	35.7%	
Private including HMO	44.9%	38.8%	44.5%	
Self-pay/no-charge/others	14.6%	16.5%	14.8%	
Non-elective admission	65.4%	75.2%	66.0%	<0.001
Bed size of the hospital				<0.001
Small	11.7%	12.8%	11.7%	
Medium	25.9%	24.7%	25.8%	
Large	62.5%	62.4%	62.5%	
Location/teaching status of the hospital				<0.001
Rural	10.5%	11.4%	10.6%	
Urban non-teaching	37.6%	34.8%	37.5%	
Urban teaching	51.8%	53.8%	51.9%	
Hospital region				<0.001
Northeast	18.2%	19.7%	18.3%	
Midwest	21.6%	27.4%	21.9%	
South	38.9%	35.4%	38.7%	
West	21.4%	17.6%	21.1%	
B. Comorbidities				
Alcohol abuse	3.6%	7.0%	3.8%	<0.001
Smoking	13.1%	30.5%	14.1%	<0.001
Drug abuse	6.7%	13.5%	7.1%	<0.001
Congestive heart failure	0.6%	1.3%	0.6%	<0.001
Hypertension	8.1%	18.9%	8.8%	<0.001
Diabetes, uncomplicated	3.3%	7.0%	3.5%	<0.001
Diabetes with chronic complications	0.8%	2.2%	0.9%	<0.001
Dyslipidemia	2.5%	7.1%	2.7%	<0.001
Obesity	6.9%	12.4%	7.2%	<0.001
Peripheral vascular disorders	0.3%	0.6%	0.3%	<0.001
Pulmonary circulation disorders	0.4%	0.8%	0.4%	<0.001
Renal failure	1.5%	3.2%	1.6%	<0.001
Valvular heart disease	0.6%	1.2%	0.6%	<0.001
Previous MI/PCI/CABG	0.4%	1.0%	0.4%	<0.001

**Figure 1:**
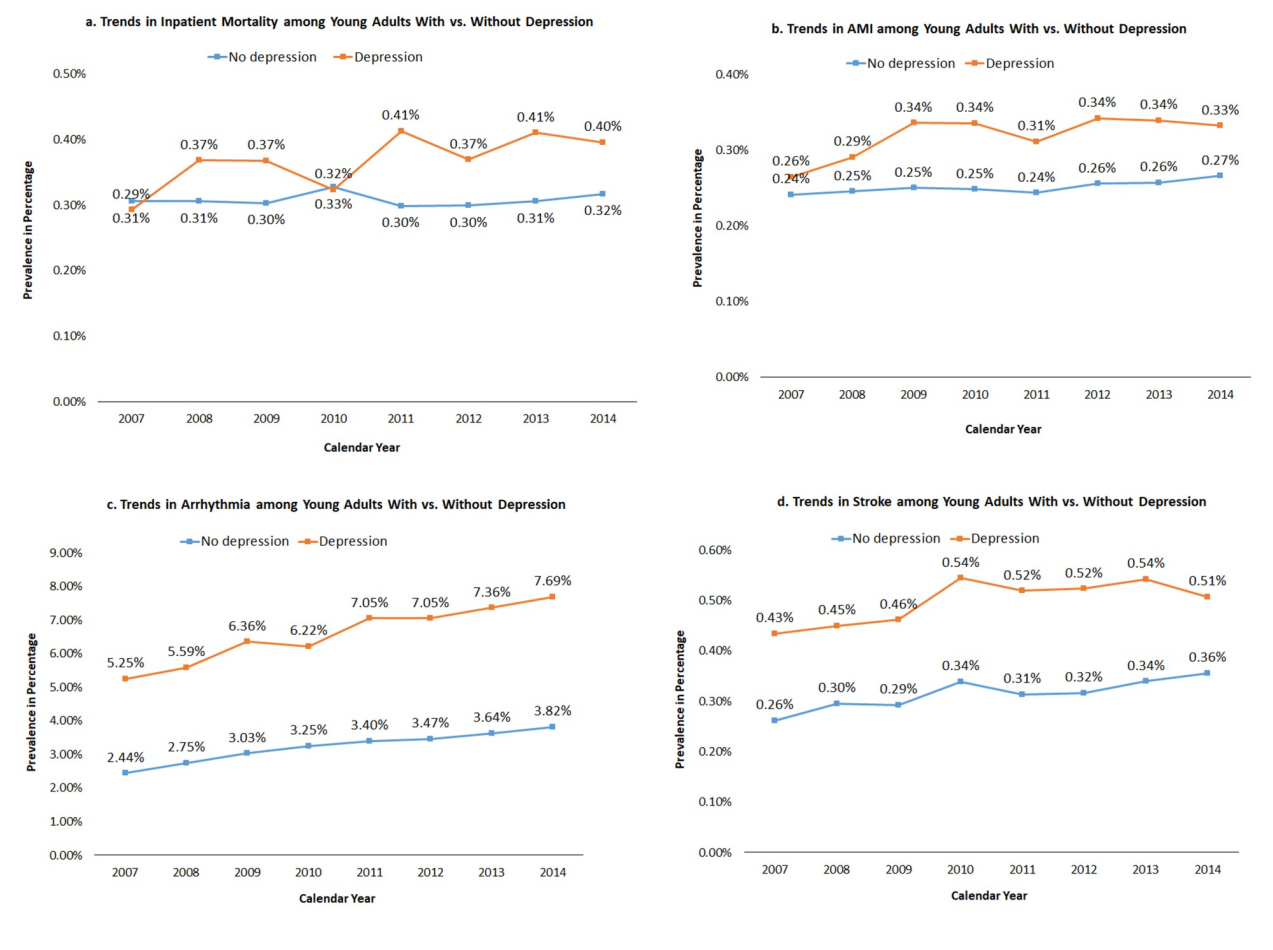
Trends in inpatient mortality, acute myocardial infarction, arrhythmia, and stroke among young adults with vs. without depression a. Trends in all-cause in-hospital mortality with vs. without depression; b. trends in acute myocardial infarction with vs. without depression; c. trends in arrhythmia with vs. without depression; d. trends in stroke with vs. without depression P<0.001 (all trends) AMI: acute myocardial infarction

We observed significantly rising trends in mortality rates among depressed inpatients from 2007 to 2014; however, the mortality rates remained stable in those without depression. The prevalence of AMI remained stable over the eight years irrespective of the state of depression whereas a steadily rising trend in the prevalence of arrhythmia was noticed, with a significantly steeper hike in depressed individuals. There was a marginally rising trend in stroke prevalence during the study period, with an interesting peak in prevalence during 2010 (Figures 1a-1d).

**Figure 2:**
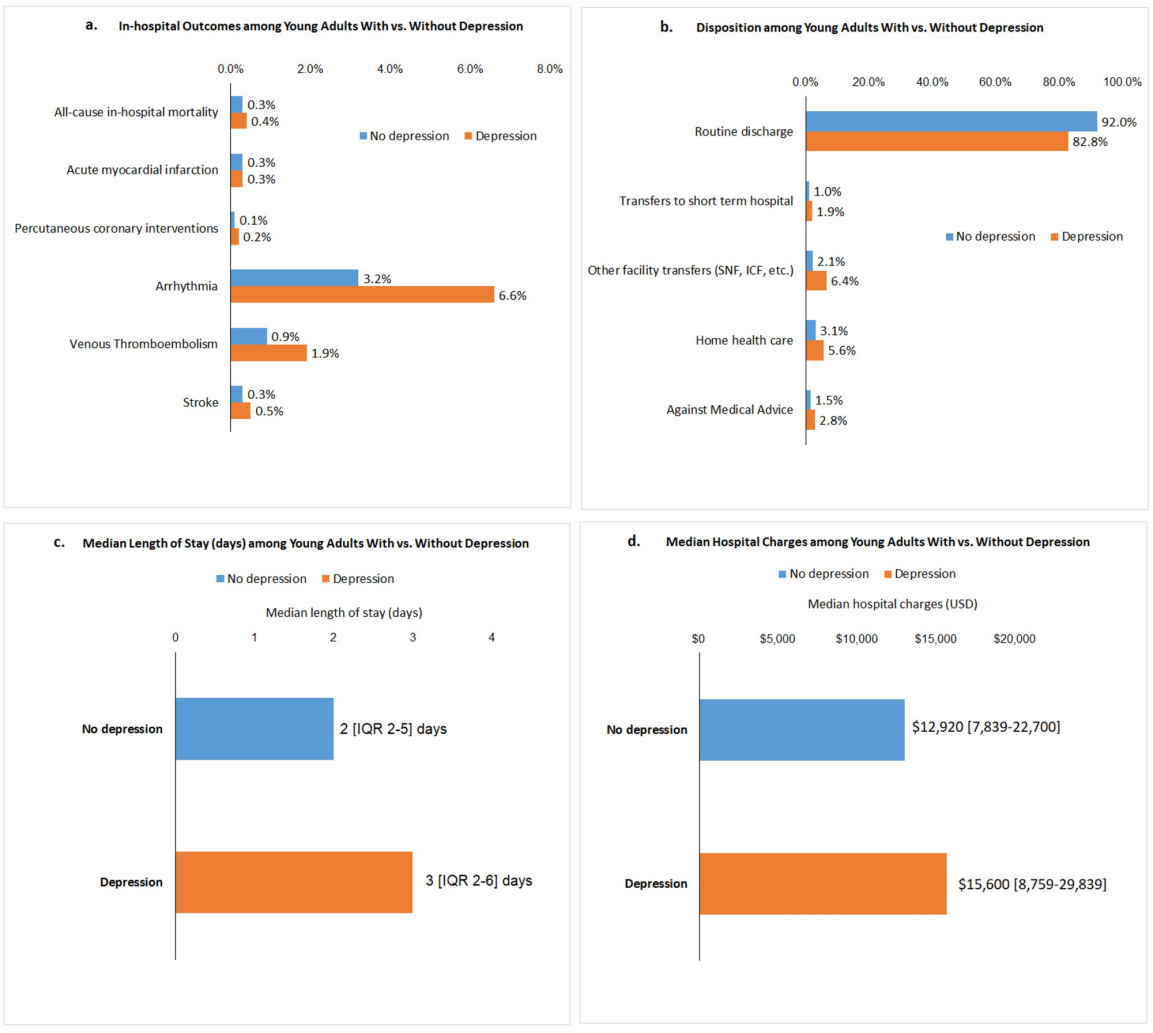
Inpatient outcomes, median length of stay (days), median hospital charges, and disposition among young adults with vs. without depression a. in-hospital complications with vs. without depression; b. disposition of patients with vs. without depression; c. mean length of stay (days) with vs. without depression; d. mean hospital charges (USD) with vs. without depression

On comparing the hospitalization outcomes in both the groups, the depressed group had significantly higher percentage of all-cause mortality (0.4% vs. 0.3%), PCI (0.2% vs. 0.1%), arrhythmia (6.6% vs. 3.2%), VTE (1.9% vs. 0.9%), and stroke (0.5% vs. 0.3%). When compared with the no-depression group, a higher percentage of patients in the depression group were either transferred to short-term hospital stays (1.9% vs. 1.0%) or other facilities (skilled nursing facility, intermediate care facility, 6.4% vs. 2.1%). A higher proportion of patients in the depression group left the hospital against medical advice compared to the non-depression group (2.8% vs.1.5%). The median LOS (days) (three vs. two days) and charges of hospitalization ($15,600 vs. $12,920) were higher in the cohort with depression (Figures 2e-2h). Comorbidities independently predicted extended LOS (>3 days) and higher hospitalization charges (>$15,600) in young adults hospitalized with depression (Table 2).

**Table 2:** Predictors of prolonged length of stay (days) and higher hospital charges in hospitalizations among young adults with depression *P-value of <0.05 indicates statistical significance OR: odds ratio; CI: confidence interval; LL: lower level; UL: upper level; MI: myocardial infarction; PCI: percutaneous coronary intervention; CABG: coronary artery bypass grafting Note: multivariable regression models were adjusted for baseline demographics, type of admission, median household income quartile for patients’ Zip Code, payer status, hospital location/teaching status, hospital bed size and hospital region, and pre-existing comorbid conditions and positive medical history

Variable	Length of stay (>3 days)				Hospital charges (>$15,600)			
	OR	95% CI		P-value*	OR	95% CI		P-value*
		LL	UL			LL	UL	
Age in years at admission	1.00	1.00	1.00	<0.001	1.02	1.02	1.02	<0.001
Male vs. female	0.77	0.76	0.77	<0.001	0.89	0.88	0.89	<0.001
African American vs. white	1.20	1.19	1.21	<0.001	1.15	1.14	1.16	<0.001
Hispanic vs. white	1.01	1.01	1.02	0.002	1.34	1.33	1.35	<0.001
Asian or Pacific Islander vs. white	1.16	1.13	1.19	<0.001	1.16	1.13	1.18	<0.001
Native American vs. white	1.06	1.04	1.09	<0.001	0.85	0.83	0.88	<0.001
Elective vs. non-elective admission	1.02	1.01	1.02	<0.001	1.48	1.47	1.49	<0.001
Comorbidities								
Alcohol abuse	0.93	0.93	0.94	<0.001	0.99	0.98	1.00	0.041
Rheumatoid arthritis/collagen vascular diseases	1.36	1.33	1.38	<0.001	1.53	1.50	1.56	<0.001
Congestive heart failure	1.39	1.36	1.43	<0.001	1.48	1.45	1.52	<0.001
Chronic pulmonary disease	1.08	1.07	1.08	<0.001	1.17	1.16	1.18	<0.001
Diabetes, uncomplicated	1.14	1.13	1.16	<0.001	1.30	1.28	1.31	<0.001
Diabetes with complications	1.72	1.69	1.75	<0.001	1.38	1.36	1.41	<0.001
Drug abuse	1.08	1.07	1.09	<0.001	1.04	1.03	1.04	<0.001
Hypertension	1.04	1.03	1.05	<0.001	1.25	1.24	1.26	<0.001
Hypothyroidism	1.08	1.07	1.09	<0.001	1.14	1.13	1.15	<0.001
Liver disease	1.33	1.32	1.35	<0.001	1.63	1.61	1.66	<0.001
Fluid and electrolyte disorders	1.59	1.58	1.60	<0.001	1.79	1.78	1.80	<0.001
Metastatic cancer	2.35	2.26	2.44	<0.001	2.75	2.65	2.86	<0.001
Other neurological disorders	1.13	1.12	1.14	<0.001	1.28	1.27	1.30	<0.001
Obesity	1.28	1.27	1.29	<0.001	1.35	1.34	1.36	<0.001
Peripheral vascular disorders	1.93	1.86	2.00	<0.001	2.33	2.24	2.42	<0.001
Pulmonary circulation disorders	2.42	2.34	2.50	<0.001	2.70	2.61	2.79	<0.001
Renal failure	1.19	1.17	1.21	<0.001	1.41	1.39	1.43	<0.001
Solid tumor without metastasis	1.72	1.65	1.79	<0.001	1.87	1.80	1.95	<0.001
Valvular heart disease	1.27	1.24	1.30	<0.001	1.41	1.38	1.45	<0.001
Dyslipidemia	0.78	0.77	0.78	<0.001	1.18	1.16	1.19	<0.001
Previous MI/PCI/CABG	0.77	0.75	0.79	<0.001	1.03	1.01	1.06	0.019
Smoking	0.96	0.96	0.97	<0.001	1.13	1.12	1.13	<0.001

## Discussion

Using a nationally representative cohort, this population-based study aimed at studying the trends in the frequency of CVD among depressed young adults (18-39 years) and understanding the differences between young adults with and without depression in terms of socio-demographics, hospital characteristic, and comorbid cardiovascular conditions. The current study shows the prevalence of depression to be around 5.7% among the inpatient population aged 18-39 years and the majority of the study population consisted of females (>70%). A population-based study of Danish young adults in 2018 showed that depressive symptoms were associated with many clinical and behavioral risk factors, with a stronger association observed among females [14]. Among the hospitalized, Caucasians tend to be more depressed compared to the other ethnic groups. This finding should be viewed against the fact that Caucasians constituted the majority of the entire study population and there is a likelihood of depression being underdiagnosed or underreported among other ethnicities [15]. Non-elective admission appears to be a risk factor for depression in hospitalized young. In view of higher mortality in depressed individuals, this finding brings to light the need for measures to address depression in patients who are admitted non-electively. Admissions in the Midwestern hospitals have shown the association of depression with CVD in the current study; however, further studies are needed to look more closely into this association. Various cardiovascular and metabolic conditions have been linked to depression and our finding supports the fact that this association holds good in a large-scale population and among young hospitalized individuals [22]. Increased risk of substance abuse and obesity can lead to worse cardiovascular outcomes in young adults with depression [14,16,17].

The current study reveals the association of depression with all-cause in-hospital mortality. This finding is in line with previous studies [23]. However, the existence of many intermediate variables that influence this association should not be undermined. A significant association between depression and arrhythmia, stroke, and VTE in the current study supports the existing evidence that depression acts at a risk for many cardiovascular conditions. Association of depression with smoking, alcohol, and substance abuse can also mediate the heightened risk for cardiac conditions in young adults [14,16,20,21].

There exists a significant association between depression and transfers to other facilities. Depressed patients also tend to have a longer hospital stay and higher hospitalization charges. Comorbidities played a significant role in predicting higher LOS and hospital charges in young adults hospitalized with depression. Many previous studies have reported a similar relationship across the ages. This finding further highlights depression as a strong predictor of poor outcomes and increased healthcare costs. This further stresses the need for therapeutic measures to address depression in order to achieve better overall outcomes in hospitalized patients.

We believe the large sample size of this study enhances the generalizability of our findings. However, a few possible limitations of the study include coding errors due to administrative data source, lack of data regarding the severity of depression, self-reported quality of life measures and long-term follow-up, and inability to establish a direct causal relationship between depression and cardiovascular events. Data regarding the history of child abuse and neglect, drug abuse, physical activity, prescribed medications, adherence to treatment plan, more recent life stressors, availability of mental health services, and outcomes related to treatment vs. lack of treatment of depression were not available.

## Conclusions

Concisely, there was a constant rise in in-hospital mortality and cardiovascular events among depressed young adults across the study period. This could possibly be due to either a direct association of depression or increased comorbid risk factors. This observation further strengthens the need to address mental health issues in young, especially those with cardiovascular risk factors, through a multidisciplinary approach.

## References

[1] GBD 2017 Disease and Injury Incidence and Prevalence Collaborators (2018). Global, regional, and national incidence, prevalence, and years lived with disability for 354 diseases and injuries for 195 countries and territories, 1990-2017: a systematic analysis for the Global Burden of Disease Study 2017. Lancet.

[2] Jiang W, Alexander J, Christopher E (2001). Relationship of depression to increased risk of mortality and rehospitalization in patients with congestive heart failure. Arch Intern Med.

[3] Nicholson A, Kuper H, Hemingway H (2006). Depression as an aetiologic and prognostic factor in coronary heart disease: a meta-analysis of 6362 events among 146 538 participants in 54 observational studies. Eur Heart J.

[4] Barefoot JC, Schroll M (1996). Symptoms of depression, acute myocardial infarction, and total mortality in a community sample. Circulation.

[5] Desai R, Singh S, Patel K, Fong HK, Kumar G, Sachdeva R (2019). The prevalence of psychiatric disorders in sudden cardiac arrest survivors: a 5-year nationwide inpatient analysis. Resuscitation.

[6] Brouwers C, Mommersteeg PM, Nyklíček I, Pelle AJ, Westerhuis BL, Szabó BM, Denollet J (2013). Positive affect dimensions and their association with inflammatory biomarkers in patients with chronic heart failure. Biol Psychol.

[7] de Jonge P, Mangano D, Whooley MA (2007). Differential association of cognitive and somatic depressive symptoms with heart rate variability in patients with stable coronary heart disease: findings from the Heart and Soul Study. Psychosom Med.

[8] Ziegelstein RC, Parakh K, Sakhuja A, Bhat U (2007). Depression and coronary artery disease: is there a platelet link?. Mayo Clin Proc.

[9] Firth J, Siddiqi N, Koyanagi A (2019). The Lancet Psychiatry Commission: a blueprint for protecting physical health in people with mental illness. Lancet Psychiatry.

[10] Schuch FB, Vancampfort D, Firth J (2018). Physical activity and incident depression: a meta-analysis of prospective cohort studies. Am J Psychiatry.

[11] Vancampfort D, Firth J, Schuch FB (2017). Sedentary behavior and physical activity levels in people with schizophrenia, bipolar disorder and major depressive disorder: a global systematic review and meta-analysis. World Psychiatry.

[12] Shah AJ, Veledar E, Hong Y, Bremner JD, Vaccarino V (2011). Depression and history of attempted suicide as risk factors for heart disease mortality in young individuals. Arch Gen Psychiatry.

[13] Meng L, Chen D, Yang Y, Zheng Y, Hui R (2012). Depression increases the risk of hypertension incidence: a meta-analysis of prospective cohort studies. J Hypertens.

[14] Dierker L, Rose J, Selya A, Piasecki TM, Hedeker D, Mermelstein R (2015). Depression and nicotine dependence from adolescence to young adulthood. Addict Behav.

[15] Woodward AT, Taylor RJ, Abelson JM, Matusko N (2013). Major depressive disorder among older African Americans, Caribbean blacks, and non-Hispanic whites: secondary analysis of the National Survey of American Life. Depress Anxiety.

[16] Edwards AC, Heron J, Dick DM, Hickman M, Lewis G, Macleod J, Kendler KS (2014). Adolescent alcohol use is positively associated with later depression in a population-based U.K. cohort. J Stud Alcohol Drugs.

[17] Hasler G, Lissek S, Ajdacic V (2005). Major depression predicts an increase in long-term body weight variability in young adults. Obes Res.

[18] (2020). HCUP Databases. Healthcare Cost and Utilization Project (HCUP). December 2019. Agency for Healthcare Research and Quality, Rockville, MD. http://www.hcup-us.ahrq.gov/nisoverview.jsp.

[19] Desai R, Patel U, Singh S (2019). The burden and impact of arrhythmia in chronic obstructive pulmonary disease: insights from the National Inpatient Sample. Int J Cardiol.

[20] Desai R, Patel U, Deshmukh A, Sachdeva R, Kumar G (2018). Burden of arrhythmia in recreational marijuana users. Int J Cardiol.

[21] Desai R, Patel U, Sharma S (2017). Recreational marijuana use and acute myocardial infarction: insights from Nationwide Inpatient Sample in the United States. Cureus.

[22] Klakk H, Kristensen PL, Andersen LB, Froberg K, Møller NC, Grøntved A (2018). Symptoms of depression in young adulthood is associated with unfavorable clinical- and behavioral cardiovascular disease risk factors. Prev Med Rep.

[23] Liu NH, Daumit GL, Dua T (2017). Excess mortality in persons with severe mental disorders: a multilevel intervention framework and priorities for clinical practice, policy and research agendas. World Psychiatry.

